# Raging elephants: effects of human disturbance on physiological stress and reproductive potential in wild Asian elephants

**DOI:** 10.1093/conphys/coz106

**Published:** 2020-01-25

**Authors:** Ruchun Tang, Wenwen Li, Di Zhu, Xiaotong Shang, Xianming Guo, Li Zhang

**Affiliations:** 1 Key Laboratory for Biodiversity Science and Ecological Engineering, Ministry of Education, College of Life Sciences, Beijing Normal University, Beijing 100875, China; 2 Research Institute of Xishuangbanna National Nature Reserve, Jinghong 666100, China

**Keywords:** Asian elephant, fecal hormone, human disturbance, reproduction, stress

## Abstract

Human disturbance has become a widespread threat to wildlife viability. The Asian elephant (*Elephas maximus*), an endangered and disturbance-prone species, is under severe threat from habitat loss and fragmentation, human–elephant conflict and poaching. Establishing connections between human disturbance, stress responses and reproduction is crucial for assessing the long-term survivability of a species and will provide critical information for conservation management. The current study investigated the effects of human disturbance on population-level stress responses and stress-related effects on reproductive potential of wild Asian elephants in Xishuangbanna Dai Autonomous Prefecture, China. We used a radioimmunoassay to measure the concentration of fecal cortisol and estradiol in 257 samples collected from five local populations at 15 sites over 4 years. Human disturbance in Xishuangbanna was quantified based on the Ecological-Niche Factor Analysis model. We found that fecal cortisol concentrations were strongly positively correlated with the degree of human disturbance and increased markedly with the expansion of tea plantations. Percentage of non-stressed individuals in a population was higher depending on the extend of undisturbed area in their home ranges. Fecal estradiol concentrations decreased significantly with increasing stress levels. Our results suggest that human disturbance poses environmental challenges to wild Asian elephant populations, and chronic exposure to human disturbance could lead to population decline. The study demonstrates the efficacy of non-invasive endocrine monitoring for further informing management decisions and developing conservation strategies.

## Teaser

The current study investigated effects of human disturbance on population-level stress responses and stress-related effects on reproduction of wild Asian elephants. The results indicate that human disturbance poses environmental challenges to wild elephant populations leading to an increased stress response and suppressed reproductive function. The study demonstrates the efficacy of non-invasive endocrine monitoring for further informing management decisions and developing conservation strategies.

## Introduction

Human disturbance, one of the principle issues of concern in conservation, has generally been considered a widespread environmental challenge and a principle threat to biodiversity (Foley *et al*., 2005; Murphy and Romanuk, 2014). Globally, expanding pastures, plantations and urban areas have encroached on a large portion of the natural habitats of wildlife. Animals adjust their distribution and behaviour to avoid contact with humans (Gill, 2007; Gaynor *et al*., 2018). However, as the human footprint expands, fewer areas are available for animals to seek spatial refuge from people (Venter *et al*., 2016). In this situation, animals in the wild must compete with humans for space and resources (Madden, 2004), which results in severe human–wildlife conflicts, and neither side benefits from such conflict.

Human disturbance could be considered as a whole group of factors, including human activity, indirect effects such as food accessibility (Martin and Réale, 2008), and potential facilitators such as geographical conditions and biological characteristics of the given species. Numerous studies have documented the effects of human disturbance on wildlife, most of which selected several disturbance factors, such as noise, roads and tourism as indicators of human disturbance (Wrege *et al*., 2010; Creel *et al*., 2013; Bhattacharjee *et al*., 2015). Studies aiming to quantify human disturbance have also been carried out. For example, Gill *et al*. (1996) calculated the extent of human disturbance as the number of disturbance events per minute of observation. Liker *et al*. (2008) quantified the degree of urbanization by scoring the occurrence of three major land-cover types and calculated the ‘urbanization score’ as the PC1 score of a principal component analysis. French *et al*. (2011) used the ‘human exposure frequency’ as a measure of human disturbance, which was calculated based on the number of days in which human presence was observed at least once divided by the number of observation days in the observation period. However, few studies have explored a method that integrates both direct and indirect effects of human activity and the geographical and biological characteristics of a given species that potentially facilitate disturbance.

In the present study, we used the ‘human–elephant conflict risk model’ (Li *et al*., 2018) to quantify the degree of human disturbance. This model was built on the ecological-niche factor analysis (ENFA) model, which has often been used to assess habitat suitability and human–wildlife conflict risk, and to predict species distribution (Le Lay *et al*., 2001; Hirzel *et al*., 2002, 2006; Enari and Suzuki, 2010; Mateo-Tomás *et al*., 2012). The ENFA model based on presence-only data shares similar principles with the principal component analysis; i.e. it extracts the primary information of each ecogeographical variable to obtain the specialization factors, which are orthogonal and contain most of the relevant information (Hirzel *et al*., 2002, 2006). Human–elephant conflict occurs when the needs of humans and elephants negatively affect each other, essentially because the expansion of human activity has occupied resources traditionally controlled by the elephants. The indirect effects and potential facilitators of human disturbance are not always observable or easy to locate. Thus, the locations where the conflict occurred are ideal ‘present points’ of human disturbance and provide large amounts of information pertaining to the consequences of disturbance.

Human disturbance may act as stressors and stimulate the hypothalamic–pituitary–adrenal (HPA) axis, resulting in elevated glucocorticoid levels. For instance, wildcats (*Felis silvestris*), giant pandas (*Ailuropoda melanoleuca*), elks (*Cervus elaphus*) and wolves (*Canis lupus*) exposed to human disturbance showed increased glucocorticoid concentrations (Creel *et al*., 2002; Owen *et al*., 2004; Piñeiro *et al*., 2012). Glucocorticoids, the end product of the HPA axis, help an organism maintain homeostasis after facing a challenge and mobilize resources to provide energy but also induce negative consequences, such as reproductive failure, stereotypic behaviour and immunosuppression (Liu *et al*., 2006; French *et al*., 2010; Strasser and Heath, 2013). In mammals, elevated cortisol (the mammalian glucocorticoid) is a risk factor for ovarian dysfunction, preterm delivery and low birth weight (Mazor *et al*., 1994; Shively *et al*., 1997; Corner *et al*., 2010). Besides, the developmental stress can have long-lasting effects on the reproductive success of future generations (Naguib *et al*., 2006; Mumby *et al*., 2015). Therefore, establishing links between human disturbance, physiological stress and reproduction is crucial for assessing the effect of human disturbance on the self-maintenance capability of wildlife populations.

The hypothalamus–pituitary–gonad (HPG) axis is a neuroendocrine system that regulates the reproductive function of the body. It is mainly composed of GnRH secreted by the hypothalamus, LH and FSH secreted by the pituitary and estradiol secreted by the gonad. Numerous studies have shown that excessive secretion of glucocorticoids during stress response can affect reproductive function at three levels of the HPG axis: hypothalamus (inhibition of GnRH secretion), pituitary (interfering with GnRH-induced LH release) and gonad (suppressing the stimulatory effect of gonadotropins on estradiol secretion) (D' Agostino *et al.* 1990; Baldwin *et al*., 1991; Kol *et al*., 1998; Daley *et al*., 1999; Consten *et al*., 2001; Dufourny and Skinner, 2002a,b; Gaytán *et al*., 2002). Therefore, in the context of this study, estradiol level can be used as an indicator of HPG axis dysfunction caused by long-term stress.

Asian elephants (*Elephas maximus*) are large, aseasonally breeding mammals and are endangered across their distribution (IUCN, 2019). The Asian elephant is also a first category protected species in China (State Administration of Forestry and Grassland, 2009). The wild population was estimated at between 41 410 and 52 345 individuals (Sukumar, 2003), with China maintaining 216–243 wild elephants (Zhang, 2018). The current distribution of Asian elephants in China is confined to Xishuangbanna Dai Autonomous Prefecture (Xishuangbanna), Lincang City and Pu’Er City (Zhang *et al*., 2015). More than half of the wild Chinese population inhabits Xishuangbanna, and the ratio of age classes of elephants in Xishuangbanna was 9.25:16.29:29.96:44.49 (calf: juvenile: sub-adult: adult). (Wang *et al.*, 2018; Zhang, 2018). Xishuangbanna is currently one of the most productive regions for rubber and tea in China, leading to severe habitat loss and fragmentation and other damage such as pollution (Liu *et al*., 2017). Accordingly, human–elephant conflict in Xishuangbanna has been increasing in both severity and frequency. Managing human disturbance requires an accurate evaluation of that disturbance and a better understanding of endocrine indicators of disturbance and how disturbance affects reproduction and survival. Our objective was to test the hypothesis that human disturbance could act as environmental stressors to wild Asian elephant populations. We investigated the links between the degree of human disturbance, cortisol concentrations (which correspond to physiological stress) and estradiol concentrations (which correspond to reproductive function). In addition, we hypothesized that prolonged exposure to human disturbance would impair reproductive potential, which would be associated with increased cortisol concentrations.

## Materials and methods

### Study area

**Figure 1:**
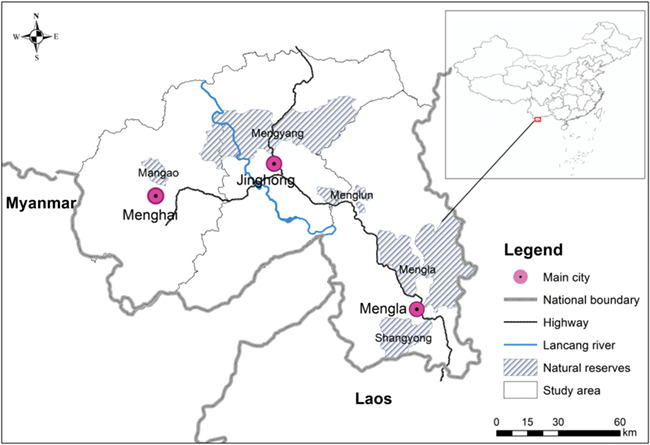
Study area: Xishuangbanna Dai Autonomous Prefecture.

The 19 100 km^2^ Xishuangbanna, Yunnan Province (99°58′–101°50′E, 21°09′–36′N) is in the southwest of China, bordering Myanmar to the South and Laos to the southeast, and is close to Thailand (Fig. 1). Xishuangbanna is warm and moist all year round, with an annual mean temperature of 15.1–21.7°C and an annual rainfall of 1193–2491 mm. The wet season lasts from May to October, and the dry season lasts from November to April of the next year. The Xishuangbanna National Nature Reserve, with a total area of 2425 km^2^, is in Xishuangbanna and consists of five sub-protected areas, which are geographically discontinuous: Mengyang, Shangyong, Mengla, Menglun and Mangao. Currently, the wild Asian elephants living in Xishuangbanna are only distributed in Menghai County, Mengyang, Shangyong and Mengla.

### Animals and sample collection

The wild Asian elephants included in this study were from populations distributed in multiple sites of Xishuangbanna, including the protected areas of Mengyang, Shangyong and Mengla, and some adjacent non-protected areas. The size of the Asian elephant populations in Xishuangbanna was estimated to be between 106 and 117 (Zhang, 2018). Besides, elephants in Pu’Er sometimes migrate between Xishuangbanna and Pu’Er (Zhang *et al*., 2015).

The study was conducted from 2014 to 2017, and 257 fresh fecal samples were collected from 15 sites (Table 1). The 15 sampling sites were: Mengwang (MW), Yegutang (YGT), Xintianba (XTB), Mengpeng (MP), Nanping (NP), Yexianggu (YXG), Shanghongshahe (SHSH), Shanghuibian (SHB), Dalongha (DLH), Zhilong (ZL), Konggeliudui (KGLD), Mangang (MG), Zhongshan (ZS), Jingne (JN) and Sunhuan (SH). We collected fresh fecal samples in the wet season of each year, the sampling periods were April to May in 2014, May in 2015, July to August in 2016, and May to June in 2017. We initiated sampling once defecation was observed by patrols. Given that sun exposure changes hormone concentrations (Wong *et al*., 2016), we collected most samples primarily during the morning and early afternoon (09.00–13.00 h) when sunlight was not too strong.

**Table 1:** Respective sample size and individual identification result at 15 sampling sites.

Sampling site	2014			2015		2016									2017
	MW	YGT	XTB	MP	NP	YXG	SHSH	SHB	DLH	ZL	KGLD	MG	ZS	JN	SH
Sample size (257)	9	6	18	10	13	8	15	4	9	43	16	17	26	20	43
Effective individual (161)	6	3	10	6	8	3	9	3	5	27	5	16	20	12	28

We measured the diameter of each sample for age estimation (Reilly, 2002; Morrison *et al*., 2005). The shape of a dung bolus is similar to that of a cylinder with slightly elliptical ends. We measured the long and short axes of the elliptical ends, and took the mean of these two measures as the diameter for a bolus. We used the rearranged Von Bertalanffy growth equation (Von Bertalanffy, 1938) to predict mean age (*t*) as a function of bolus diameter (*L*):

$$t=\left({t}_0-\left(1/k\right)\right)\times \left(\mathrm{In}\left(1-L/{L}_{\infty}\right)\right),$$

where *t*_0_ is the theoretical age at which dung diameter is 0, *k* is the Brody growth coefficient and *L*_∞_ is the is the theoretical maximum size of bolus diameter. According to the parameter values (*L*_∞_, *k* and *t*_0_) estimated for Asian elephants by Reilly (2002), we regarded individuals with dung diameter greater than 12.67 cm as adults.

**Figure 2:**
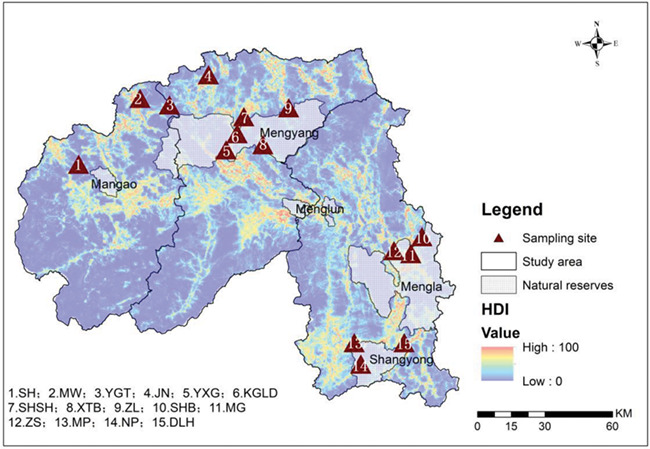
Map showing the sampling sites (filled triangles) and spatial distribution of human disturbance in Xishuangbanna.

After measurement, the entire fecal dropping was thoroughly mixed to ensure an even distribution of hormones (Wasser *et al*., 1996), sub-samples were then collected from several locations of the dropping and stored at −20°C until hormone analysis. For DNA analysis, 5 g of dung was collected for each sample peeled from the outer layer containing exfoliated intestinal epithelial cells and preserved in 95% ethanol. New gloves and collection tubes were used for each sampling to avoid cross contamination. Those samples were stored at ambient temperature and transported to the laboratory for cryopreservation as soon as possible. The positions of all fecal samples were recorded using GPS (Fig. 2).

### Hormone extraction

We used a vortexing extraction method described in Wasser *et al.* (2000), with some modification based on Wasser *et al.* (2010). The vortexing extraction was proved to produce consistent and high (~85–100%) recoveries of endogenous fecal steroids in species such as grizzly bear, elk and elephant (Wasser *et al*., 2000). In brief, feces were freeze-dried, pulverized and sifted to remove plant materials. Dry feces were extracted (0.8 g) in 5 ml of 95% ethanol by vortexing for 30 min and centrifuging at 2250 g for 20 min. Supernatants were recovered and centrifuged again. Supernatants were nitrogen-dried, and extracts were diluted with 1 ml of saline and stored at −20°C until analysis.

### RIA procedure and validation

Fecal cortisol and estradiol were measured by ^125^I-labelled radioimmunoassay kits (Beijing North Biotechnology Institute, Beijing, China) using the manufacturer’s instructions. The antibodies in these two assays were raised in rabbits. For both assays, intra- and inter-assay coefficients of variation were ˂10% and ˂15%, respectively. All hormone measures were expressed per gram of dry weight to control the effects of dietary changes on hormone excretion rates (Wasser *et al*., 1993).

For cortisol RIA, standards (50 μl, range 10–500 ng/ml) or fecal extracts (50 μl) were pipetted in duplicate into tubes, and combined with 100 μl ^125^I-cortisol tracer and 100 μl antibody. In addition, we set up tubes containing 100 μl distilled water, 100 μl ^125^I-cortisol tracer and 50 μl ‘0’ standard but no antibody to estimate non-specific binding and total radioactivity. All the tubes were incubated at 37°C for 45 min. 500 μl charcoal–dextran (0.125% Norit-A Charcoal, 0.0125% dextran in 0.1 mol PBS, PH 7.0) was then added to separate antibody-bound and free cortisol, and the tubes were incubated at room temperature for an additional 15 min, followed by centrifugation at 4°C for 15 min at 2013 g. The precipitate was measured for 2 min in a XH-6080 radioimmunoassay γ counter (Shanghai Heyi Instruments and Meters Co., Ltd, Shanghai, China). The sensitivity of the assay was 2 ng/ml. The assay was validated by demonstrating parallelism between standard curves and serial dilution of fecal extracts (*R* > 0.99).

For estradiol RIA, standards (100 μl, range 10–1000 pg/ml) or fecal extracts (100 μl) were pipetted in duplicate into tubes, and combined with 100 μl ^125^I-estradiol tracer and 100 μl antibody. We set up tubes containing 100 μl distilled water, 100 μl ^125^I-estradiol tracer and 100 μl ‘0’ standard but no antibody to estimate non-specific binding and total radioactivity. All the tubes were incubated at 37°C for 1.5 h, separation of antibody-bound and free estradiol was achieved with the addition of 500 μl charcoal–dextran (0.125% Norit-A Charcoal, 0.0125% dextran in 0.1 mol PBS and PH 7.0) and an incubation period of 15 min at room temperature, followed by centrifugation at 4°C for 20 min at 2130 g. The precipitate was measured for 2 min in a XH-6080 radioimmunoassay γ counter (Shanghai Heyi Instruments and Meters Co., Ltd, Shanghai, China). The assay sensitivity was 5 pg/ml. The estradiol radioimmunoassay was validated by demonstrating parallelism between serial dilutions of fecal samples and standards (*R* > 0.99).

### Genetic identification of individuals

Owing to the low DNA quantities in feces (Taberlet *et al*., 1996), DNA extraction was performed twice for each fecal sample following the protocols described by Du (2017). Six microsatellite markers for Asian elephants and four markers for African elephants (*Loxodonta africana*) published by Fernando *et al*. (2001), Cai *et al*. (2008), Comstock *et al*. (2010), Nyakaana and Arctander (2010), Nyakaana *et al*. (2010), and Kongrit *et al*. (2010) were tested to identify the best markers for achieving consistently successful amplification. We selected ten markers (EMU04, EMU07, EMU11, EMU12, EMU14, EMU15, EMX2, FH60, FH94 and LafMS09) in our study (see Supplementary Table S1 for details). Based on a preliminary analysis of amplification products, we optimized the PCR procedures. Cycling conditions were 95°C for 15 min, 40 × (95°C for 1 min, 58°C for 1 min and 72°C for 1 min), and 72°C for 15 min. The 10 μl reaction volume consisted of 5 μl HotStar Taq mix (2.5 units HotStar Taq DNA polymerase, 1× PCR Buffer, 1.5 mM MgCl_2_, 200 μM of each dNTP) (QIAGEN), 2.4 μl ddH_2_O, 2 μl DNA, 0.2 μl forward primer, 0.2 μl reverse primer and 0.2 μl bovine serum albumin (10 μg/μl). According to the multiple tubes approach (Taberlet *et al*., 1996), each DNA sample was amplified twice, and a total of four repeated amplifications of each fecal sample were used for cross-validation of genotyping. The allele sizes were determined using GeneScan™ 1200 LIZ Size Standard (Applied Biosystems, USA). The fragment sizes were analysed using Applied Biosystems 3730-XL (Applied Biosystems, USA) and scored using GeneMapper v.4.0. Individual identification was carried out in CERVUS v.3.0.

Samples were typed as heterozygous at one locus when both alleles appeared at least twice amongst the four replicates and as homozygous when identical alleles were observed in all replicates (Bellemain *et al*., 2005). If neither of those two cases applied, then the samples were amplified another four times for reanalysis. If more than two alleles appeared, we treated those samples as cross contaminated and discarded them. We considered genotypes from different samples as representing an identical individual when all alleles at all loci were identical. However, if there was only one mismatch for one allele at one locus, then we assumed that those samples belonged to the same individual (Bellemain *et al*., 2005; Solberg *et al*., 2006). In statistical analysis, the number of effective individuals was regarded as the true sample size (Table 1), and we averaged the hormone data from the same individual.

### Assessment of human disturbance

We divided Xishuangbanna into a 0.004 km^2^ (200 × 200 m) grid network to assess human disturbance variables such as frequency of tea gardens and farms, land-use types, distance to main roads and countryside trails from the centre of the 0.004 km^2^ grids, and ecogeographical variables such as elevation, slope, aspect, distance to mainstreams, tributaries, reserves and elephant distributions from the grids. These 12 variables were selected after being tested for spatial autocorrelation, in which, one of the two variables with a correlation coefficient higher than 0.60 was excluded from the final model. Elevation, slope and aspect were extracted from the ASTER GDEM (30 × 30 m spatial resolution, downloaded from Chinese Geospatial Data Cloud, http://www.gscloud.cn). The vegetation map and the digitized data on rivers, villages and roads were provided by the Yunnan Institute of Forestry Inventory and Planning and the Administration of Xishuangbanna National Nature Reserve. Elephant distributions referred to the feces locations recorded using GPS. All variables were processed using ArcGIS v.10.1 (ESRI, CA, USA) to develop the spatial grid data of the same range, resolution and geographical coordinates, and all individual layers were then imported into the Biomapper v.4.0 software (URL: http://www2.unil.ch/biomapper), in which we used the ENFA model to calculate the human disturbance index (HDI). We took the locations of 245 villages where human–elephant conflict occurred between 2011 and 2015 as ‘presence points’, the information of which was provided by China Pacific Insurance Company. The HDI calculated using the ENFA model ranged from 0 to 100; then, we labeled the study area with different human disturbance levels, viz. none (0–19), low (20–39), medium (40–59), obvious (60–79) and high disturbance (80–100). The HDI of each sampling site was calculated as the average within a radius of 1.5 km centred on the feces.

### Statistical analysis

The Kruskal-Wallis *H* test was used to conduct pairwise comparisons of fecal cortisol concentrations (FCC) amongst sampling sites and amongst disturbance levels (data were not normally distributed). We also compared FCC amongst sampling sites using general linear model (GLM). The model was developed by setting FCC as the dependent variable and including the following independent variables as factors: sampling year, sampling site and age group (factor with two levels: adult and non-adult). The initial model included two-way interaction between sampling site and age group. Non-significant factors and interaction were removed from the models, removing the one with largest *P* value in each step. We used Tukey’s Honestly Significant Difference *post-hoc* tests for pairwise comparisons between sampling sites. To remove confounding effects of other variables, tests were conducted on residuals calculated from the final model. Pearson Correlation was performed between mean cortisol values and HDI scores, residual cortisol values and HDI scores, as well as mean cortisol values and disturbance factors (distance to main roads and countryside trails) (data had a normal distribution). The relationships between average cortisol and estradiol concentrations, average cortisol concentrations and disturbance factors, such as frequency of farms and tea gardens, were assessed using Spearman Correlation (data were not normally distributed). The exponential regression model was used to determine the variation of cortisol concentrations with reference to frequencies of tea gardens. All the statistical analyses were carried out using SPSS v.20.0 (IBM, USA). In all analyses, *P* < 0.05 was considered to be significant.

## Results

### HDI

About 22.77% (4349.08 km^2^) of the prefecture area was deemed to be disturbed (Fig. 2): low, 1495.11 km^2^ (34.38%); medium, 1280.87 km^2^ (29.45%); obvious, 1069.45 km^2^ (24.59%); high, 503.65 km^2^ (11.58%) of the total disturbed area. Most of the disturbed areas were composed of agricultural land (67.51%) including garden plots and farms, in sharp contrast to forests (27.97%). The HDI and different disturbance factors at 15 sampling sites are listed in Table 2. NP, MW, YGT and XTB were considered undisturbed for elephants. Low disturbance was found at YXG, SHSH, SHB, DLH, MP and ZL. SH, KGLD, MG, ZS and JN were recorded as medium-disturbance areas (Table 2).

**Table 2:** Human disturbance factors and HDI of sampling sites in Xishuangbanna.

Sampling site	Frequency of tea garden	Frequency of farm	Distance to main road (m)	Distance to countryside trail (m)	HDI
NP	0	0.045	10 573	1486	11
MW	0	0.13	5606	1462	14
YGT	0	0.0197	12 662	994	15
XTB	0	0.0365	10 360	1010	17
YXG	0.019	0.03	730	789	23
SHSH	0.0156	0.0067	1726	1209	25
SHB	0	0.069	5133	686	30
DLH	0	0.14	2089	656	36
MP	0	0.011	898	749	36
ZL	0.0753	0.033	13 722	1502	37
SH	0.032	0.37	974	370	43
KGLD	0.062	0.0246	1729	516	46
MG	0	0.071	690	428	47
ZS	0.0273	0.015	4429	833	47
JN	0.0314	0.24	5774	290	52

### Stress response to human disturbance

**Figure 3:**
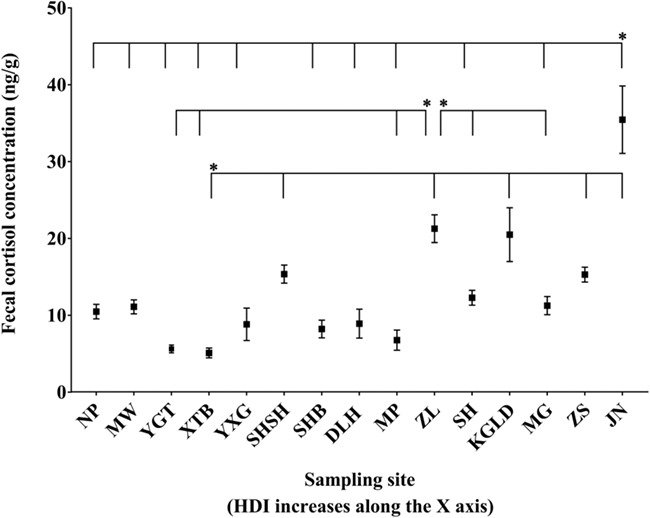
Differences in FCC of elephants between differently disturbed sampling sites. The figure shows mean ± SE values. Horizontal lines connecting different sites indicate significant pairwise differences between sampling sites. Asterisk * indicates the site from which the others differ.

**Figure 4:**
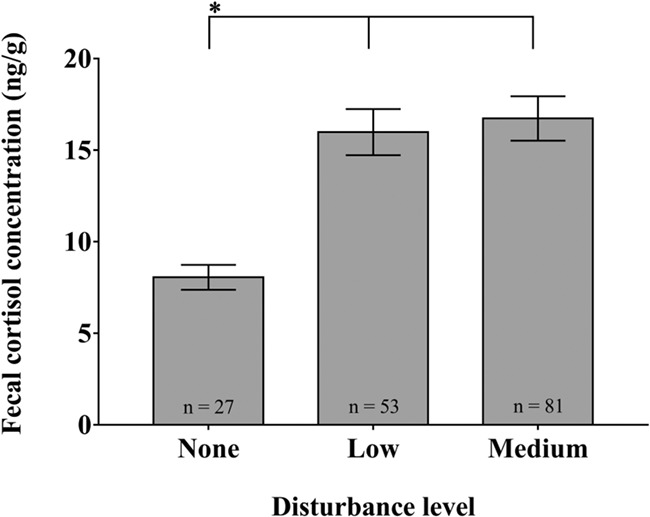
Average FCC and different levels of human disturbance. Horizontal lines indicate significant differences, asterisk * indicates the level from which the others differ. Results are mean ± SE.

FCC was not significantly affected by age in this study but differed significantly between sampling years and there was no significant interaction between sampling site and age group (Table 3). FCC differed significantly amongst sampling sites (d*f* = 14, *χ*^2^ = 96.091, *P* = 0.000) with the feces collected at JN showing the highest mean FCC values (35.42 ± 4.39 ng/g) and the feces collected at XTB showing the lowest mean FCC values (5.08 ± 0.63 ng/g). Specifically, the average FCC in feces collected at more-disturbed sites was generally higher than those at less-disturbed sites (in all cases of the 18 significant pairwise differences) (Fig. 3). The average FCC of elephant populations were significantly lower when elephant populations were in the undisturbed areas than when they were in the disturbed areas (d*f* = 2, *χ*^2^ = 27.496, *P* = 0.000) (Fig. 4).

**Table 3:** Results of the final GLM analysing variation in fecal cortiol concentration.

Dependent variable	Predictor variable	d*f*	*F*	*P*
Fecal cortisol concentration	sampling site	11	11.67	˂0.0001
	sampling year	3	21.33	˂0.0001

The initial model included age group as explanatory variable (d*f* = 1, *F* = 2.66, *P* = 0.105), which is excluded from the final model and not shown. Final model: adjusted *R*^2^ = 0.527, d*f* = 160.

**Figure 5:**
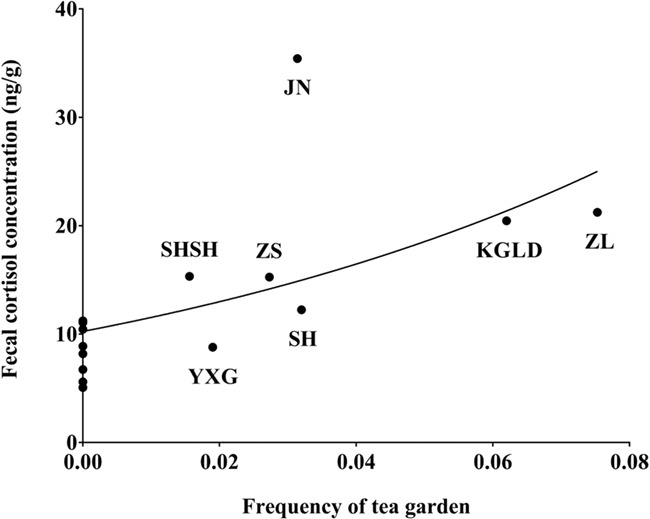
Relationship between mean FCC of elephant populations and tea garden frequencies at different sampling sites. The points on the *Y* axis from top to bottom are: MG, MW, NP, DLH, SHB, MP, YGT and XTB (see Table 2 for sampling site abbreviations).

The average FCC of elephant populations showed an upward trend with proximity to countryside trails and increase in farm frequency, although the differences were not significant. A strong positive correlation was found between average FCC and frequency of tea gardens (rho = 0.779, *n* = 15, *P* = 0.001). The average FCC showed a significant exponential pattern as a function of tea garden frequency (*y* = 15.58*^x^* + 8.67_,_ adjusted *R*^2^ = 0.481, d*f* = 13, *P* = 0.002) (Fig. 5).

**Figure 6:**
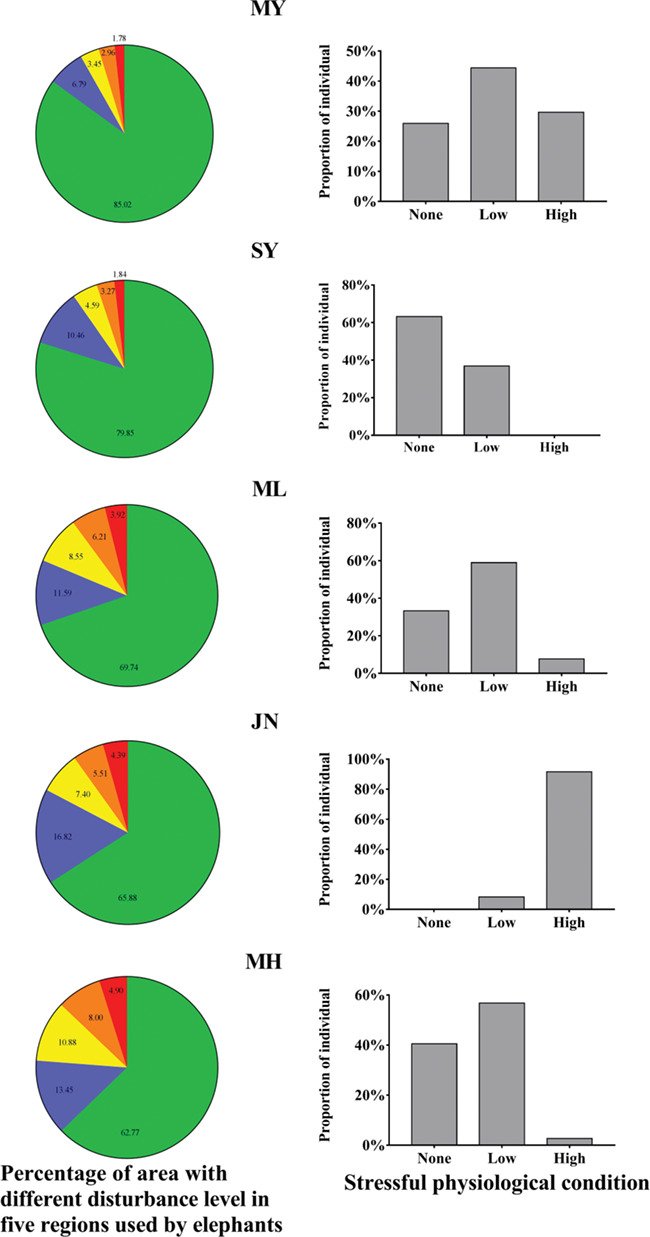
Percentage of differently disturbed areas and proportion of individuals under different stressful conditions in five regions used by different local populations. Green: no disturbance, blue: low disturbance, yellow: medium disturbance, orange: obvious disturbance, red: high disturbance. The cortisol concentrations were divided into three levels of stressful physiological conditions: (1) no stress (0, 10), (2) low stress [10, 20) and (3) high stress (≥20). The sample size for MY, SY, ML, JN and MH was 54, 13, 45, 12 and 37, respectively.

For local populations in SY, ML and JN, the proportion of highly stressed individuals in populations increased with decreasing percentage of non-disturbed area in their home ranges. In contrast, non-stressed condition benefited from the expansion of undisturbed area (Fig. 6). Particularly, almost all individuals in the JN population (11 of 12), which roamed within a region with the largest percentage of disturbed area, were at a high level of stress compared with elephant populations living in regions with larger undisturbed areas. Nevertheless, similar patterns were not observed in MY and MH.

**Figure 7:**
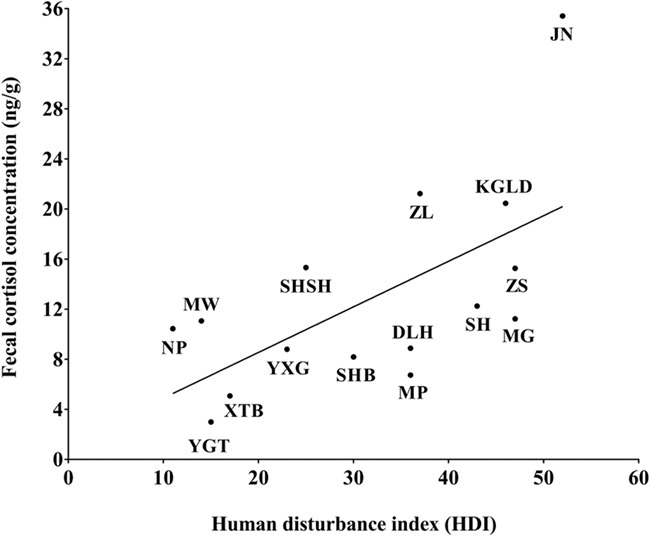
Relationship between mean FCC of elephant populations from different sampling sites and sampling site HDI (see Table 2 for sampling site abbreviations).

The linear relationship between the HDI of sampling sites and the average FCC of elephant populations verified the above results. There was a strong positive correlation between average FCC and HDI (*r* = 0.620, *n* = 15, *P* = 0.014). For the linear regression model predicting FCC, adjusted *R*^2^ = 0.319 (*P* = 0.017) (Fig. 7). It is necessary to point out that we calculated residuals for FCC from the final GLM, correlated residual FCC with HDI and obtained the similar result, although the relationship was not significant (*r* = 0.463, *n* = 15, *P* = 0.082) (Supplementary Fig. S1). We believe that the correlation test between FCC and HDI is more appropriate in analysing whether the variation in FCC is related to human disturbance. Although the results of the final GLM showed that FCC differed significantly between sampling years (FCC in samples collected in 2016 were significantly higher), but this did not mean that FCC had undergone interannual changes. During the study, each site was sampled only once and samples came from different populations. Of the nine sampling sites in 2016, only one was located in the undisturbed area, four in the low-disturbance area and four in the medium-disturbance area. The variation in FCC between years was actually caused by varying degrees of human disturbance at the sampling sites we visited in 2014–2017. Therefore, the effect of sampling year was actually unnecessary to be controlled, otherwise the effects of human disturbance will be underestimated.

### Reproductive endocrines

**Figure 8:**
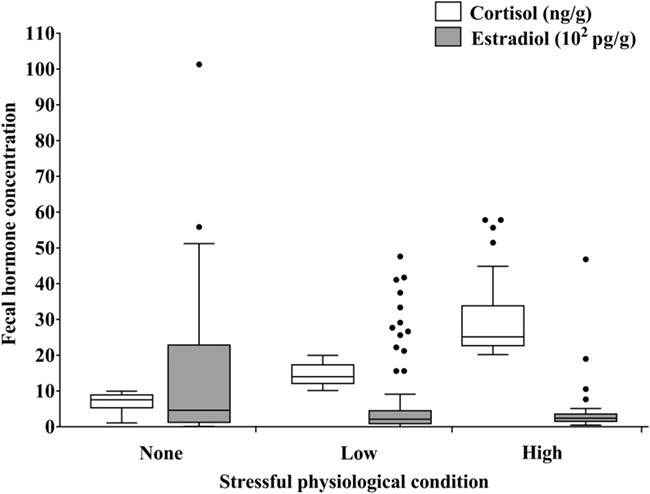
Fecal estradiol and cortisol concentrations at 14 sampling sites, summarized in box and whisker plots, associated with stressful physiological conditions (stress level division as in Fig. 6). Outliers are shown as points. The sample size for non-stress, low-stress, and high-stress conditions was 52, 75 and 31, respectively.

Fecal estradiol concentrations differed significantly amongst sampling sites (d*f* = 14, *χ*^2^ = 114.05, *P* = 0.000). There was a significant negative correlation between FCC and fecal estradiol concentrations with elephant populations under more stressful physiological conditions showing reduced estradiol concentrations (rho = −0.159, *n* = 158, *P* = 0.046) (Fig. 8). Samples collected at SHB were excluded from the estradiol analysis because these samples were from a bull-only group.

## Discussion

In the present study, we tested whether the degree of human disturbance affects physiological stress and reproductive potential in wild Asian elephant populations living in Xishuangbanna, China. We conducted the entire study during the wet seasons to avoid seasonal rhythms in cortisol secretions caused by changes in food abundance between the dry and wet seasons (Marcilla *et al*., 2012). We found that populations differed significantly in FCC, with cortisol concentrations being generally higher in feces collected at disturbed sites than that collected at undisturbed sites. The average FCC of the population at the most disturbed site (JN) was 3.4 times that of the population at the least disturbed site (NP). The strong positive correlation between HDI and FCC supports the hypothesis that human disturbance can pose environmental challenges to wild elephant populations and cause an enhanced population-level stress response.

Two results provided further evidence that human disturbance provokes an increase in cortisol concentrations. At the fine spatiotemporal scale, the FCC of elephant populations increased significantly with increased frequency of tea gardens within a 1.5 km radius. Land-use change is a well-known disturbance factor threatening wildlife population viability, and may act as a stressor by reducing resource availability, increasing competition, and altering disease stressors associated with changes in population density (McCallum and Dobson, 2002; Acevedo-Whitehouse and Duffus, 2009; Mbora and McPeek, 2009; Brearley *et al*., 2013). Over the last 40 years, the natural forests in Yunnan Province have severely decreased and fragmented in response to increasing tea and rubber plantations encroaching on previously forested land, with the greatest loss of forests occurring in Xishuangbanna. However, two major conservation policies in the late 1990s (the Natural Forest Conservation Program and the Sloping Land Conservation Program) and the decreasing price of rubber led to tea plantations becoming the fastest growing industry. It has been reported that the area of tea gardens increased by 1956 km^2^ from 2005 to 2014, 85% of which was converted from forests, while a small portion of rubber plantations (210 km^2^) was converted to forests (Liu *et al*., 2017). Given that the area covered by farms, rivers and cities from 2005 to 2014 remained relatively stable, we have good reason to believe that the continuous development of tea plantations was the main driver of land-use change in Xishuangbanna in recent years. It seems probable that the strong effect of human disturbance on FCC is mediated, at least in part, by land-use change.

At the broad spatiotemporal scale, the proportion of non-stressed individuals in populations increased with the extent of undisturbed area in their home ranges, indicating that the level of stress condition at the population level reflects the overall human disturbance within the home ranges. Our samples were not evenly distributed through the home ranges of elephants because the use of tracking collars on elephants has not been approved in China, which makes the real-time telemetry of elephant locations impossible. However, elephant herds are able to trek long distances, and their daily movements usually range from a few kilometers to 20 km, even distances of 90–180 km have been observed (Sukumar, 2003). Therefore, the samples from several sites may not fully reflect how elephants were disturbed on a larger scale.

To compensate for the uneven distribution of samples and provide a comprehensive picture of the effects of human disturbance on FCC, we analysed the correlation between the composition of different degrees of disturbance and the percentage of stressful individuals in five local populations. In addition, the fecal glucocorticoids levels reflect the stress levels experienced by animals ~6–12 h before defecation instead of the pulsatile secretion pattern of HPA axis (Windle *et al*., 1998; Harper and Austad, 2000). This explains why the FCC was relatively low in some samples collected from sites where the HDI was high. However, we did not observe this similar pattern in MY and MH. There are three possible explanations for this discrepancy. The first is that the Sixiao Expressway, which segregates the MY reserve into east and west parts, acts as an environmental stressor on the local population, inducing stress, and thereby obscuring any differences in FCC that may have existed between populations from MY and the other four regions. Some of our sampling sites in MY reserve (YXG, KGLD and SHSH) were located near the expressway, and these samples exhibited relatively high cortisol levels. The results of some previous studies on the stress responses of animals to roads are consistent with our results (Andrews, 1990; Reijnen *et al*., 1995; Wasser *et al*., 1997; Forman and Alexander, 1998; Bhattacharjee *et al*., 2015). Second, we found from this research that elephants in MY preferred to live near human settlements rather than in the vast undisturbed core areas, which increased the frequency of interactions with people, and thereby made elephants more exposed to human disturbance. Our field survey showed that many trees in the core areas of MY reserve grew so high that they limited the growth of the shrub layer, which provided food for elephants (Lin *et al*., 2016). We speculate that the limited food accessibility in the core areas drove elephants to search for food near human settlements. The third is that the low intragroup competition may contribute to the less stressful conditions in the MH population. According to our field survey, there is only one group of elephants in MH, consisting of 18 individuals, suggesting that the population density and intragroup competition are relatively low. Some authors regard competition as a precipitating role in enhancing stress responses (Foley *et al*., 2001; Hing *et al*., 2014).

The results of our study showed that estradiol and cortisol concentrations were negatively correlated with each other. Although we cannot exclude the potential sex-related differences in fecal estradiol output, they are unlikely to interfere with our results because of the particular social structures of Asian elephants (Sukumar, 1989; Dublin, 1996). Adult females and their offspring form the family group, which is the basis of the family unit. Females remain with the family group throughout their lives, whereas males leave their natal groups after sexual maturity. Males that disperse from their families either live independently or form bull-only groups with other males. We excluded the feces from the bull-only group and independent males from the statistical analysis. The influence of male calves is slight at the population level and can be ignored.

Numerous studies have shown that chronic stress has the potential to suppress reproduction by acting on the hypothalamus, pituitary or the gonads (Saketos *et al*., 1993; Tilbrook *et al*., 2000; Consten *et al*., 2001; Dufourny and Skinner, 2002b; Gaytán *et al*., 2002; Maya-Núñez and Conn, 2003). Moreover, reproduction is influenced strongly by external stressors. For instance, a recent study on timber elephants in Myanmar demonstrated that elephants born in the high stress season, which corresponds to intense workload, experienced faster reproductive ageing (Mumby *et al*., 2015). Similarly, African elephants manifested declining progesterone concentrations during the harshest portion of the dry season, when water and food availability were at their lowest (Foley *et al*., 2001). These observations, combined with our results, suggest that human disturbance caused suppressed reproductive potential in elephant populations, which was mediated by elevated stress hormones.

Several conservation and management recommendations can be drawn from our study. First, noninvasive measures of fecal hormone concentrations can be used to monitor stress and reproductive function across a large number of wild elephant populations. These methods are particularly valuable for monitoring forest populations, where difficult terrain and dense vegetation restrict observations. Such combined measures can help to determine whether populations are threatened by current human disturbance, and which populations are in greater need of protection. Second, noninvasive endocrine monitoring is a reliable tool to evaluate the efficacy of present management decisions. For instance, the contradiction between the vast undisturbed area in MY and the large percentage of stressed individuals in the population, combined with the fact that the MY population abandoned the undisturbed area and chose to live near human settlements, could reflect the irrationality of functional area planning in the reserve. Finally, the fact that elephants mounted a strong stress response to factors related to land-use change reminds managers to balance economic development with habitat protection.
